# Skin prick test to foods in childhood atopic eczema: pros and cons

**DOI:** 10.1186/1824-7288-39-48

**Published:** 2013-07-31

**Authors:** Carlo Caffarelli, Arianna Dondi, Carlotta Povesi Dascola, Giampaolo Ricci

**Affiliations:** 1 Pediatric Unit, Azienda Ospedaliera- universitaria, Allergology and Immunology Unit, Department of Clinical and Experimental Medicine, University of Parma, Parma, Italy; 2 Pediatric Unit, Department of Gynecological, Obstetric and Pediatric Sciences, S.Orsola-Malpighi Hospital, University of Bologna, Bologna, Italy; 3 Dermatology Unit, Department of Specialistic, Diagnostic and Experimental Medicine, S.Orsola-Malpighi Hospital, University of Bologna, Bologna, Italy

**Keywords:** Atopic dermatitis, Atopic eczema, Skin prick test, Food allergy

## Abstract

Skin prick tests are the first investigation in allergy diagnostics and their use is described in all the guidelines on atopic eczema. However, the clinical usefulness of skin prick tests is the subject of great debate. On the one hand, skin prick tests allow the identification both of individuals at risk for food allergy and of the allergen inducing the eczematous flare. On the other hand, when performed by a non-specific specialist, positive skin prick tests to foods may wrongly lead to prolonged elimination diets, which may induce nutritional deficiencies and perhaps loss of tolerance to the avoided foods. Furthermore, skin prick tests increase health costs. A consensus on this topic has not yet been reached. Considering the diversity of clinical stages in which it occurs, atopic eczema presentation should be the starting point to determine whether or not skin prick tests should be carried out.

## Introduction

Childhood atopic eczema (AE) is a common chronic inflammatory skin disease. Whether food allergens play a pathogenetic role in inducing exacerbations of AE is a longstanding matter of debate. Some clinicians consider AE to be strongly related to food allergy and perhaps healed by food avoidance [1-3], but others substantially deny such a relationship [4], limiting investigations for food allergy to severe cases in infancy who do not respond to treatment [5]. Clinical history may provide useful hints to suspect the offending food, even if parents generally overestimate the frequency of food reactions [6]. IgE tests are performed to identify causative food allergens, but the ultimate mean for ascertaining food allergy is the oral provocation challenge. Among the tests used in clinical practice for detecting IgE-mediated sensitivity, skin prick tests (SPTs) are commonly performed as the first step since they are easy to do, cause almost no trauma to the infant or child, are less expensive compared with serum specific IgE (sIgE) antibodies and the results are quickly ready to support the possible diagnosis of IgE-mediatd food allergy [7-9]. Furthermore, it has been found that negative SPT results exclude immediate reactions to the suspected food [8]. A wheal diameter for common foods above certain levels has been proposed to make oral food challenge unnecessary because it has a high positive predictive value [10,11].

During oral food challenge, exacerbations of AE can be delayed up to several hours or even days after ingestion of the relevant food, suggesting a non IgE-mediated mechanism [12,13]. Therefore, it is controversial whether SPTs are useful for diagnosing or screening allergy in children with AE.

In the present article, both the advantages and disadvantages of performing SPT in children with AE have been reviewed (Table 1).

**Table 1:** Pros and cons of executing skin prick test for food allergens in children affected by atopic eczema

**Pros of executing SPT**	**Cons of executing SPT**
• Identification of triggers of flare	• Lack of standardized skin prick test technique
• Diagnosis of associated clinical hypersensitivity reactions to foods	• Dietary restrictions based only on SPT results leading to: - loss of tolerance
• Prediction of reaction at first ingestion of egg and peanuts	- nutritional problems
• Younger children	• Mild disease
• Moderate-to-severe disease	• Not specific for the diagnosis of AE
• Recognition of children at risk for respiratory atopic diseases	• Increase health cost

## Pros of executing SPT in children with AE

Several advantages have been attributed to SPT execution over non-execution: detection of children with associated food allergy, identification of foods causing AE flares, screening of children at risk for clinical hypersensitivity reactions to food, early identification of patients at risk for allergic respiratory disease (Table 1).

### Detection of foods that exacerbate AE

There is some evidence that, in a subgroup of patients, eczematous lesions can be significantly worsened by food intake. After an oral food challenge, some children develop a rapid-onset itchy rash, either isolated or as part of a systemic reaction [12,13]. Other children may present a flare of AE some hours or days later [12,13], which may be isolated in about 12% of instances or preceded by a non eczematous immediate reaction in 45% of cases [12]. Moreover, it has been noted that AE improves when the foods responsible for immediate reactions are eliminated from the diet [13]. For instance, when hen’s egg is avoided in children with positive sIgE to egg, AE improves [14].

Taking into consideration that AE improves with age and allergy to the most common allergenic foods in infants (cow’s milk and hen’s egg) is often outgrown during childhood [3], SPT to food may be less helpful beyond infancy. However, in older children as well as in adults there are some reports that there is a late-onset exacerbation of AE following challenge with birch pollen-related foods [15,16].

In the diagnostic work-up for identifying foods that are responsible of eczematous flare-up, history is often unreliable and SPT diagnostic accuracy is low [12,17]. The improvement of skin symptoms following an elimination diet is not enough to ascertain that a particular food is the culprit of AE. Oral provocation challenge remains the standard for the diagnosis [12,18-22]. When no immediate reactions are observed, the administration of the food should be continued for at least two days.

### Identification of eczematous children with associated food allergy

In 40% to 90% of eczematous children, tests for IgE-mediated hypersensitivity to foods result as positive [23-25]. However, a positive SPT does not mean that the child has a clinical immediate hypersensitivity to that food. Any positive IgE test to foods is irrelevant if not in agreement with a possible clinical history and confirmed by an oral provocation test. This is warranted not only to prevent possibly harmful reactions, but also to avoid useless diets. The latter point is of particular importance when the implicated food is essential for nutrition, such as cow’s milk in very young babies. Positive IgE tests to foods confirmed by the onset of immediate reactions upon oral food challenge have been reported in 30–60% of children with AE [13,17,26], mostly in the case of moderate-to-severe AE [27-30]. An oral food challenge is not necessary when a positive SPT to a specific food is associated with a clear-cut history of an anaphylactic reaction to that food. In food-dependent exercise-induced anaphylaxis, SPT is helpful in identifying foods whose intake before physical exercise might provoke an anaphylactic reaction [31,32].

### Screening of children at risk for immediate reactions to food

Children with a positive SPT to hen’s egg [33] or peanuts [34] and who had never previously ingested these foods are at risk for immediate reactions at the first intake. On the other hand, a negative SPT result to a food is useful to exclude with high accuracy that the child will not have immediate reactions to its intake [8].

### Early identification of patients at risk for allergic respiratory disease

Atopic sensitization and the development of allergic asthma and/or rhinitis is very common among AE patients, and its early detection and prevention are a major part of AE global management [1,9]. It has been shown that infants with the early development of IgE sensitization to food allergens have an increased risk for later development of inhalant sensitization in childhood [35,36]. In children with AE a positive SPT results to egg at 1 year of age was associated with subsequent asthma at 4 years of age [37] and of respiratory atopic diseases such as rhinoconjunctivitis and asthma at 6 years of age [38-40]. On the other hand, house dust mites allergy is considered, per se, to be an aggravating factor in AD patients, becoming increasingly relevant during chidhood and adolescence.

## Cons of executing SPT in children with AE

There are some arguments suggesting that not carrying out SPT is better than executing them in children with AE. These points mainly include poor SPT standardization, long elimination diets with a subsequent risk of severe reactions because of loss of tolerance to the avoided foods and high health costs (Table 1).

### Lack of standardized SPT technique

Most commercially available food allergen extracts for SPT as well as the technique for skin testing are not standardized [41]. Food reagents are usually classified according to weight by volume in the absence of characterization of potency, allergenic molecules, or protein content [42]. Therefore, sensitivity of food extracts may vary from company to company [43]. Serum food sIgE levels obtained by different assays are not equivalent, too [44]. Thus, published data on the diagnostic accuracy of SPT or sIgE antibodies to foods should be considered only relevant for that study and hardly comparable with the results of other studies. Skin testing with fresh food seems to enhance sensitivity [45] and specificity [46]. SPT with molecular food allergen may have increased sensibility. However, studies comparing SPTs with food extracts and those with natural foods or with allergen components in children with AE are warranted to extend previous findings. Such studies should take into account that many patients who have allergic reactions to fresh foods can tolerate the same food when it is cooked or baked [47].

### Dietary restrictions in children with AE

There are some old low-quality studies supporting the efficacy of an exclusion diet in unselected patients with AE [48]. When the diet was effective, SPT reactions were not useful to predict which children would have had an improvement of the skin lesions [29]. Overall, a cost/benefit analysis suggests that it is not recommended to perform SPT in children with mild-to-moderate AE and on a non-restriction diet [49].

Another issue is that, when SPT results show a sensitization against one or more foods in children with AE, elimination of these foods from the child’s diet is often carried on for several months before the patient is referred for oral food challenge. Only a short-term (no more than 2–3 weeks) diet, with a subsequent intake of the food in order to ascertain its etiologic role, is acceptable. Some cases have been reported in which, after a long period of exclusion diet, children affected by AE had anaphylactic reactions to cow’s milk that had never occurred previously [50,51], suggesting that the diet itself might have favored the loss of immunologic tolerance and the onset of food allergy. This implies that, under these circumstances, the reintroduction of the food should be always planned in a hospital setting, despite the inconveniences for children or parents and the costs for the structure.

Another problem is that children on a prolonged diet might undergo nutritional deficiencies, including failure to thrive and kwashiorkor [52] when they are not supervised by a dietitian.

### AE diagnosis and clinical course

No specific marker for the diagnosis of AE has been reported. The diagnosis mainly relies on a combination of clinical features [53]. Positive SPT results are listed by Hanifin and Rajka [54] among the minor criteria for AE diagnosis. However, there is no evidence that positive SPT can play a role for this diagnosis [55]. The sensitization to food allergens can follow the occurrence of AE, but can also precede and predict AE onset [56]. It is unclear whether the number of positive SPT results is associated with AD severity [29,55,57-62] Contrasting data have been provided also on the association between sensitization and AE persistence [60,61].

Longitudinal studies have shown that early long-term sensitization may be associated with more severe AE [63].

## Concluding remarks

AE therapy is based on the use of emollients and topical or, less frequently systemic corticosteroids. There is evidence that, in a subgroup of patients, foods can trigger late onset eczematous flares. Elimination of identified food allergens has been shown to provide improvement of AE symptoms, but combined with good skin care and pharmacotherapy when needed. AE, however, has causes that are also not related to food allergies. Patients who respond well to skin care treartment with minimal topical steroids treatment are not likely to benefit from dietary intervention when no history of immediate food allergic reactions are reported.

In children with AE, SPT for foods can be proposed to detect an IgE-mediated sensitization to foods and predict the risk of immediate reactions after their ingestion. A short-term diet and a well-defined reintroduction plan should be offered. A prolonged elimination diet should be followed only for foods which have been demonstrated by oral challenge to be responsible for adverse reactions.

A number of matters continue to be unclear. Are SPTs with fresh food or molecular allergens useful in identifying children with AE flare-up caused by foods? To what extent can the evidence obtained by AE improvement in infants following an elimination diet be transferred to different age groups? Further studies are necessary to clarify the unmet needs of SPTs to foods in children with atopic eczema, namely the prediction of response to elimination diet in different age group children, the identification of foods which exacerbate AE, the diagnostic accuracy of SPT with fresh foods, extracts or allergen components, and the performance of cost-effectiveness studies.

Finally, we believe that the results of SPT in young children with AE may be helpful in identifying or excluding an associated food allergy and in recognizing which patients have an atopic background and thus require a follow up also targeted to the early detection and prevention of allergic asthma and/or rhinitis. SPT results should not be interpreted by a physician who was not trained in allergy as inaccurate clinical practice may lead to prolonged and potentially harmful restriction diets.

## Competing interest

The authors declare that no conflict of interest exists concerning the present manuscript.

## Authors’ contribution

CC performed study design, literature search, preparation of first draft, draft revision. AD performed preparation of the first draft, literature search, revision. CPD performed literature search, revision of the drafts, preparation of the final version. GR performed study design, literature search, preparation of first draft, draft revision. All authors read and approved the final manuscript.
